# The relationship between 2019-nCoV and psychological distress among parents of children with autism spectrum disorder

**DOI:** 10.1186/s12992-021-00674-8

**Published:** 2021-02-25

**Authors:** Luxi Wang, Dexin Li, Shixu Pan, Jinhe Zhai, Wei Xia, Caihong Sun, Mingyang Zou

**Affiliations:** 1 Department of Children’s and Adolescent Health, Public Health College, Harbin Medical University, Harbin, 150081 China; 2 Department of Children Psychology, Zhuhai Maternal and Child Health Care Hospital, Zhuhai, 519001 China

**Keywords:** Autism spectrum disorder, 2019 novel coronavirus disease, Parents, Anxiety, Depression

## Abstract

**Objectives:**

The psychological distress caused by COVID-19 may be pronounced among the parents of children with autism spectrum disorder (ASD). This study aimed to investigate psychological distress among parents of children with ASD during the COVID-19 pandemic.

**Methods:**

A total of 1764 parents of children with ASD and 4962 parents of typically developing (TD) children were recruited. The participants completed an online survey which contained demographic information, the impact due to COVID-19 crisis, resilience, coping styles, anxiety and depression. Hierarchical linear regression was used to assess the contributions of these variables to anxiety and depression.

**Results:**

After adjusting for demographic variables, the following factors were associated with parents’ anxiety and depression symptoms: (i) Whether or not the participants had a child with ASD; (ii) resilience; (iii) coping strategies, and; (iv) the impact due to COVID-19. Among these, the psychological stress caused by COVID-19 played the most important role in parental anxiety (*β* = 0.353) and depression (*β* = 0.242) symptoms. Parents of children with ASD had lower levels of resilience and positive coping, and used more negative coping strategies than parents of TD children. Among all participants, 8.0 and 24.2% of parents had symptoms of anxiety and depression, respectively. Compared to parents of TD children, more parents of children with ASD exhibited symptoms of anxiety and depression (12.2% vs. 6.6%; 31.0% vs. 21.7%, respectively).

**Conclusions:**

During the COVID-19 pandemic, parents experienced varying levels of anxiety and depression, particularly, parents of children with ASD. More specific attention should be paid to parental mental health and long-term effective intervention programs, that are targeted towards parents of children with ASD, and such programs should be promoted around China in the wake of the COVID-19 crisis.

**Supplementary Information:**

The online version contains supplementary material available at 10.1186/s12992-021-00674-8.

## Background

The 2019 novel coronavirus disease (2019-nCov or COVID-19) refers to pneumonia caused by severe acute respiratory syndrome coronavirus 2 (SARS-CoV-2), which has spread rapidly around the world and poses a serious threat to global public health [1]. On January 30, 2020, the World Health Organization (WHO) listed the COVID-19 pandemic as a “Public Health Emergency of International Concern”. The Chinese government rapidly implemented a series of effective public health interventions to control the COVID-19 outbreak (including but not limited to, the timely treatment of patients, isolation and quarantine, travel bans, a ban on mass gatherings, social distancing measures, and the disinfection of public places). From SARS, H1N1, MERS to COVID-19, public health emergencies not only place individual lives at risk, but they also adversely affect the mental health of individuals, eliciting, for example, fear, anxiety, depression and worries during, and even after, the outbreak of such epidemics [2–5]. The COVID-19 pandemic, which is of an unparalleled magnitude and intensity, presents a more serious challenge to all members of the community, especially to parents, who are stuck at home, compelled to juggle their work and family life, who have to go the extra mile to balance their work arrangements, finances, and childcare [6]. The task of keeping children busy and safe at home is, in and of itself, a daunting challenge.

Autism spectrum disorder (ASD) represents a collection of heterogeneous neurodevelopmental disorders with deficits in reciprocal social communication and social interactions, and restricted repetitive patterns of behavior [7]. Most individuals with ASD may need care or assistance from their parents and families across their lifespans. ASD affects around 1% of children in mainland China, which is comparable to western countries [8]. A recent report published by the Autism and Developmental Disabilities Monitoring Network demonstrated that the prevalence of ASD has reached 1.85% (i.e., one in 54) among children [9]. However, there are no specific and effective medications for ASD treatment, which is mainly based on rehabilitative training to improve core social and communicative skills impairments, as well as other possible co-comorbidities. Early, comprehensive, and intensive behavioral intervention is recognized as an effective approach for improving the prognosis of children with ASD [10, 11].

Unfortunately, school closures and home confinement are now widespread as part of China’s response to the COVID-19 pandemic. Although these measures are commendable and necessary, the resulting disruption to the daily routines of children with ASD can cause incredible hardship. Long-term changes to training schedules can lead to developmental regression and the loss of skills that have been acquired during the school program, as well as to increased abnormal behaviors and emotional problems. The lack of access to adequate and professional home settings poses a serious threat to their physical and mental health, adversely impacting the effectiveness of their rehabilitation, and even the quality of life of the entire family. Most parents of children with ASD have to manage their children’s emotional, functional, and behavioral problems at home and without assistance. The child’s noncompliance, disturbed mood or irritability, and increasingly maladaptive behaviors will leave parents frustrated and lacking confidence in their parenting abilities. The high demands placed on parents of children with ASD take a toll, leading to both physiological and mental fatigue [12]. Moreover, family financial loss, social quarantine, greater amounts of caregiving time, decreased parenting self-efficacy, inadequate support services, the absence of clarity regarding duration of the lockdown situation, and uncertainty about the children’s future during the pandemic, all contribute to an accumulation of stresses that are experienced by ASD parental groups. Notably, high parental stress can, in turn, have a negative impact on the affected children’s psychological well-being, and exacerbate ASD-related behaviors and symptoms, creating a vicious circle [13]. Therefore, it is necessary to pay greater attention to addressing the impact that the COVID-19 epidemic has on mental health, which is equally important for parents and children.

This study aimed to investigate the level of the impact of COVID-19, resilience, coping strategies, anxiety and depression symptoms among parents of children with ASD, and to assess the factors related to parents’ psychological distress during the COVID-19 pandemic. Our results provide evidence which highlights effective strategies and intervention measures that can be used by government officials who can formulate relevant policies to mitigate the psychological distress of parents of children with ASD.

## Methods

### Participants

In this cross-sectional study, which was conducted in three provinces (Heilongjiang, Henan, and Fujian) in northern, central, and southern China, data were collected during the COVID-19 pandemic from March to April, 2020. Eligible participants included parents who raised a child with ASD. The diagnosis of ASD was obtained from two independent specialist clinicians, and it was based on the diagnostic criteria outlined in the DSM-V [7]. It could also be verified by referring to the Disable Person’s Federation registry system. The parents of typically developing (TD) children from normal nursery, primary, junior, and senior schools were recruited as controls. All procedures were carried out an adequate understanding and each participant provided online informed consent prior to the commencement of the study. This study was approved by the Institutional Review Board of Harbin Medical University for Medical Sciences.

### Procedure

Participants completed a self-report questionnaire which consisted of six sections that addressed demographic information, the impact due to COVID-19 crisis, the Connor-Davidson Resilience Scale (CD-RISC), the Simplified Coping Style Questionnaire (SCSQ), the Self-rating Anxiety Scale (SAS), and the Self-rating Depression Scale (SDS). The questionnaire was distributed by means of an online survey platform (i.e., Questionnaire Star, Changsha Ranxing Science and Technology, Shanghai, China). The survey was carried out over a period of 50 days. The invitations provided the participants with a QR code to access to the online questionnaire. The teachers in special schools or regular schools sent the invitations to parents of children with ASD and to parents of TD children, respectively. A uniform rubric was used at the beginning of the questionnaire to explain the purpose and significance of the questionnaire, as well as the method to be used to complete it. All participants were invited to provide their online informed consent prior to data collection. All information was anonymized to ensure confidentiality. Each questionnaire item could not be repeated or ignored, otherwise, it was not deemed valid. The same IP address could be used only once to complete the questionnaire. A participant’s questionnaire was considered invalid in the following cases: (a) items of the scales answered with identity or regularity; (b) “not applicable” responses; (c) the time spent on the entire questionnaire was less than five minutes. A total of 8133 questionnaires were distributed and 6726 valid questionnaires were retrieved, resulting in an effective recovery rate of 82.7%.

### Demographic information

Demographic information included the following: personal demographics (i.e., province, parents’ gender, age), socio-economic status (i.e., health status, education level, and occupation), child’s characteristics (i.e., child’s age, gender), family variables (i.e., only one child in the family, parents’ marital status, and family income per month).

### Impact due to COVID-19 crisis

The impact section was designed to collect data related to the particular conditions brought about by the COVID-19 crisis, which included the following: parents’ identity (a member of the general public; member of a public group in isolation due to confirmed or presumed cases; being a close contact; due to travel history; front-line staff: medical staff, CDC technical, or the police, etc.), changes in relationship (with parents, lovers, children and friends), changes in physical exercise (duration, intensity, and frequency), changes in daily diet (appetite and regularity), changes in household income, and a self-designed questionnaire. The Psychological Stress from the COVID-19 Questionnaire (PSCQ) was used to evaluate behaviors and parents’ perceptions (see Appendix 1). This questionnaire was prepared in consultation with relevant experts and scholars, and it was revised on the basis of a preliminary survey involving a small sample. It consisted of 15 items, which were rated using a Likert-type scale which ranged from 0 (not at all) to 3 (very frequently) according to the frequency of the listed events, and scores were summed to produce a total score. Higher scores indicated a higher level of psychological stress. The researchers then sub-divided psychological stress into four categories, in accordance with previous studies [14, 15]: category 1, if psychological stress ≤ P25; category 2, if P25 < psychological stress ≤ P50; category 3, if P50 < psychological stress ≤ P75; category 4, if psychological stress > P75. In this sample, the internal consistency reliability of the scale reached 0.88.

### Connor-Davidson resilience scale (CD-RISC)

The CD-RISC was developed to describe an individual’s psychological feelings during the previous month. It consists of 25 items, which are categorized into three dimensions, i.e., tenacity, strength, and optimism. Each item is scored using a five-point Likert-type scale which ranges from 0 to 4 according to the frequency of symptoms. The total score ranges from 0 to 100. Higher scores indicate stronger psychological elasticity. The Chinese version of the CD-RISC also had good internal consistency [16]. In this study, the Cronbach’s alpha for the scale was 0.96.

### Simplified coping style questionnaire (SCSQ)

The SCSQ is a 20-item self-assessment questionnaire consisting of two dimensions, i.e., positive coping (1–12 items) and negative coping (13–20 items). Four possible answers are allowed (i.e., “never”, “occasionally”, “sometimes”, and “always”), and the participants are requested to rate each item from 0 to 3 based on the frequency with which they use a given strategy when addressing a stressful situation or problem. Higher scores indicate a more frequent adoption of that coping strategy when faced with stress. This questionnaire had good reliability and validity in Chinese [17]. In this study, the Cronbach’s alpha for the scale was 0.87.

### The self-rating anxiety scale (SAS)

The SAS was used to measure the anxiety symptoms of parents. The SAS questionnaire contains 20 items, which are scored using a four-point Likert-type scale according to the frequency of symptoms experienced during the past week, and scores range from 1 to 4. The score of each item was calculated to obtain the raw score, and the standard score was equal to the raw score multiplied by 1.25. The cut-offs for the SAS standard scores were defined as follows: a score of less than 50 indicated no anxiety; 50–59, mild anxiety; 60–69, moderate anxiety, and; more than 70, severe anxiety [18]. The Chinese version of the scale had adequate reliability and validity. In the current study, the Cronbach’s alpha for the scale was 0.85.

### The self-rating depression scale (SDS)

The SDS comprises 20 items that are used to evaluate symptoms of depression. Participants rated each item, which was scored from 1 to 4, according to how they felt during the preceding week. The score of each item was calculated to obtain the raw score, and the standard score was equal to the raw score multiplied by 1.25. The Standard Score was classified as follows: a score of less than 50 indicated no depression; 50–59; mild depression; 60–69, moderate depression, and; greater than 70, severe depression [19]. The adequate reliability and validity of the SDS was confirmed by previous studies [20]. In the current study, the Cronbach’s alpha for the scale was 0.87.

### Statistics

Continuous variables were described as mean (M) and standard deviation (SD), and the differences between two groups were compared using independent t-tests. Categorical variables were described as frequencies with percentages, and the differences between the two groups were compared by carrying out chi-square tests. Spearman’s correlation coefficient was calculated to gain an initial insight into the interrelatedness between the study variables and to verify multicollinearity. We also examined the residuals of the regression analyses for the two outcome variables (SAS and SDS scores) to test for the assumptions of linearity, homoscedasticity, independence, and normality. Subsequently, hierarchical linear regression analysis was conducted to test the associations of potential explanatory variables and the incremental predicted variance of any given set of variables. The variables were input according to the following steps. In model 1, we input all demographic variables to control any potential confounding factors. In model 2, we added groups (families with TD children or children with ASD). In model 3, we added “resilience” and “coping strategy”. In model 4, we added “the impact due to the COVID-19 crisis”, which included the parents’ identity, relationship changes, physical exercise changes, dietary changes, income changes and psychological stress (the codes of variables are shown in Table S1). The standardized estimate (*β*), *F*, *R*^2^ and *R*^2^-change (Δ*R*^*2*^) for each model were determined. Given the multiple comparisons, the significance level was set at *P* < 0.001 (two-tailed). All statistical analyses were performed using SPSS version 21 (SPSS Inc., Chicago, IL, USA).

## Results

### Descriptive statistics

Among the 6726 participants who took part in this research, 1764 (26.2%) parents from the sample were from an ASD family and 4962 (73.8%) parents were from a TD family. A total of 3805 (56.6%) were from Heilongjiang Province, 1507 (22.4%) were from Henan Province, and 1414 (21.0%) were from Fujian Province. Most of the (84.9%) participants were mothers. Demographic details are shown in Table 1.

**Table 1:** The descriptive statistics of demographic information, impact due to COVID-19, and scores from scales or questionnaires

Variables	Total participants (*n* = 6726)	ASD family (*n* = 1764)	TDC family (*n* = 4962)	*χ*^*2*^ */ t*	*P*
Demographic characteristics					
Province, n (%)					
Heilongjiang	3805 (56.6%)	997 (56.5%)	2808 (56.6%)		
Henan	1507 (22.4%)	398 (22.6%)	1109 (22.3%)		
Fujian	1414 (21.0%)	369 (20.9%)	1045 (21.1%)	.040	.981
Parents’ gender, n (%)					
Male	1017 (15.1%)	246 (13.9%)	771 (15.5%)		
Female	5709 (84.9%)	1518 (86.1%)	4191 (84.5%)	2.572	.113
Parents’ age (years), n (%)					
20 ~ 30	1270 (18.9%)	350 (19.8%)	920 (18.5%)		
31 ~ 40	4086 (60.7%)	1038 (58.8%)	3048 (61.4%)		
41 ~ 50	1302 (19.4%)	359 (20.4%)	943 (19.0%)		
51 ~ 60	68 (1.0%)	17 (1.0%)	51 (1.0%)	3.868	.276
Parents’ health status, n (%)					
Well	6506 (96.7%)	1639 (92.9%)	4867 (98.1%)		
Diseased	220 (3.3%)	125 (7.1%)	95 (1.9%)	110.009	<.001
Parents’ education, n (%)					
Secondary school or below	2611 (38.8%)	683 (38.7%)	1928 (38.9%)		
High school or same level	2518 (37.4%)	729 (41.3%)	1789 (36.1%)		
College or same level	1441 (21.4%)	317 (18.0%)	1124 (22.7%)		
Postgraduate	156 (2.3%)	35 (2.0%)	121 (2.4%)	24.142	<.001
Parents’ occupation, n (%)					
Manual workers	2119 (31.5%)	395 (22.4%)	1724 (34.7%)		
Mental workers	2182 (32.4%)	420 (23.8%)	1762 (35.5%)		
Unemployed	1738 (25.8%)	755 (42.8%)	983 (19.8%)		
Others	687 (10.2%)	194 (11.0%)	493 (9.9%)	385.557	<.001
Child’s gender, n (%)					
Male	3819 (56.8%)	1297 (73.5%)	2522 (50.8%)		
Female	2907 (43.2%)	467 (26.5%)	2440 (49.2%)	273.251	<.001
Child’s age (years), n (%)					
Up to 3	424 (6.3%)	187 (10.6%)	237 (4.8%)		
3 ~ 6	2870 (42.7%)	801 (45.4%)	2069 (41.7%)		
6 ~ 12	2436 (36.2%)	472 (26.8%)	1964 (39.6%)		
12 ~ 18	996 (14.8%)	304 (17.2%)	692 (13.9%)	142.822	<.001
Only child in the family, n (%)					
Yes	3641 (54.1%)	1003 (56.9%)	2638 (53.2%)		
No	3085 (45.9%)	761 (43.1%)	2324 (46.8%)	7.157	.008
Parents’ marital status, n (%)					
Married	6383 (94.9%)	1641 (93.0%)	4742 (95.6%)		
Divorced or widowed	343 (5.1%)	123 (7.0%)	220 (4.4%)	17.336	<.001
Family income per month, n (%)					
< 3000	1798 (26.7%)	648 (36.7%)	1150 (23.2%)		
3000 ~ 6000	2511 (37.3%)	641 (36.3%)	1870 (37.7%)		
6001 ~ 9000	1171 (17.4%)	218 (12.4%)	953 (19.2%)		
9001 ~ 12,000	577 (8.6%)	118 (6.7%)	459 (9.3%)		
12,001 ~ 15,000	284 (4.2%)	50 (2.8%)	234 (4.7%)		
> 15,000	358 (5.7%)	89 (5.0%)	296 (6.0%)	147.960	<.001
Impact due to COVID-19 crisis					
Parents’ identity, n (%)					
General public	6305 (93.7%)	1682 (95.4%)	4623 (93.2%)		
Isolated public	38 (0.6%)	17 (1.0%)	21 (0.4%)		
Front-line staff	383 (5.7%)	65 (3.7%)	318 (6.4%)	24.348	<.001
Relationship changes, n (%)					
Increased	525 (7.8%)	160 (9.1%)	365 (7.4%)		
No change	4942 (73.5%)	1293 (73.3%)	3649 (73.5%)		
Decreased	1259 (18.7%)	311 (17.6%)	948 (19.1%)	6.423	.040
Physical Exercise changes, n (%)					
Better	887 (13.2%)	179 (10.1%)	708 (14.3%)		
No change	2250 (33.5%)	571 (32.4%)	1679 (33.8%)		
Worse	3589 (53.4%)	1014 (57.5%)	2575 (51.9%)	25.214	<.001
Dietary changes, n (%)					
Better	986 (14.7%)	209 (11.8%)	777 (15.7%)		
No change	4239 (63.0%)	1103 (62.5%)	3136 (63.2%)		
Worse	1501 (22.3%)	452 (25.6%)	1049 (21.1%)	24.705	<.001
Income changes, n (%)					
Normal income	2105 (31.3%)	416 (23.6%)	1689 (34.0%)		
Partial income	2774 (41.2%)	703 (39.9%)	2071 (41.7%)		
No income	1847 (27.5%)	645 (36.6%)	1202 (24.2%)	118.750	<.001
Psychological stress, (M, SD)					
Low	2062 (30.7%)	584 (33.1%)	1478 (29.8%)		
Relatively low	1655 (24.6%)	417 (23.6%)	1238 (24.9%)		
Relatively high	1474 (21.9%)	357 (20.2%)	1117 (22.5%)		
high	1535 (22.8%)	406 (23.0%)	1129 (22.8%)	8.695	0.034
Psychological assessment					
Resilience, (M, SD)					
Total score	70.3 ± 18.5	66.4 ± 19.5	71.7 ± 18.0	10.308	<.001
Tenacity	34.7 ± 10.3	32.7 ± 10.6	35.4 ± 10.1	9.581	<.001
Strength	24.6 ± 6.1	23.3 ± 6.5	25.0 ± 5.9	10.201	<.001
Optimism	11.0 ± 3.4	10.4 ± 3.5	11.2 ± 3.3	8.970	<.001
Coping style, (M, SD)					
Total score	35.1 ± 9.2	34.5 ± 9.6	35.4 ± 9.1	3.339	.001
Positive coping	25.1 ± 6.7	24.1 ± 7.0	25.4 ± 6.6	7.017	<.001
Negative coping	10.1 ± 4.9	10.4 ± 4.9	10.0 ± 4.9	−3.310	.001
Anxiety, (M, SD)/ n (%)	37.4 ± 8.2	38.7 ± 9.3	36.9 ± 7.7	−7.583	<.001
None	6183 (92.0%)	1548 (87.8%)	4635 (93.4%)		
Mild	427 (6.3%)	159 (9.0%)	268 (5.4%)		
Moderate	101 (1.5%)	47 (2.7%)	54 (1.1%)		
Severe	15 (0.2%)	10 (0.6%)	5 (0.1%)	65.486	<.001
Depression, (M, SD)/ n (%)	41.3 ± 11.7	43.7 ± 12.7	40.5 ± 11.2	−10.102	<.001
None	5101 (75.8%)	1218 (69.0%)	3883 (78.3%)		
Mild	973 (14.5%)	275 (15.6%)	698 (14.1%)		
Moderate	567 (8.4%)	221 (12.5%)	346 (7.0%)		
Severe	85 (1.3%)	50 (2.8%)	35 (0.7%)	110.954	<.001

*ASD* autism spectrum disorder, *TDC* typically developmental children, *M* mean, *AD* standard deviation

The parents of children with ASD had lower scores for resilience than parents of TD children in terms of both the total score and the score for all three dimensions (*P* < 0.001). Compared with parents of TD children, the total scores for “coping style” and “positive coping” were lower among parents of children with ASD, whereas the scores for “negative coping” were higher. The parents of children with ASD scored significantly higher in the SAS and SDS compared with parents of TD children (*P* < 0.001). The detection rate of anxiety and depression symptoms among total participants were 8.0% (SAS score ≥ 50) and 24.2% (SDS score ≥ 50), respectively. ASD families had a higher rate of anxiety and depression symptoms than TD families (anxiety: 12.2% vs. 6.6%; depression: 31.0% vs. 21.7%). Moreover, mothers had a higher rate of anxiety and depression symptoms than fathers either in ASD family (anxiety: 12.6% vs. 10.2%; depression: 31.7% vs. 26.4%) or in TD family (anxiety: 7.0% vs. 4.4%; depression:23.0% vs. 14.8%).

### Bivariate correlations

The bivariate correlations, which are shown in Table 2, suggested that scores of anxiety and depression were significantly correlated with “relationship changes,” “physical exercise changes,” “dietary changes,” “income changes,” “psychological stress”, “resilience” and all of its three dimensions, as well as “coping style” and all of its two dimensions (*P* < 0.01). The scores of depression and anxiety were significantly positively correlated (*r* = 0.733, *P* < 0.01). However, no relationship was found between “parents’ identity” and scores of anxiety and depression. The three dimensions (i.e., “tenacity,” “strength” and “optimize”) were highly associated with total resilience scores (*r* > 0.7, *P* < 0.01). The total scores of coping strategy” were highly correlated with “positive coping” (*r* = 0.84, *P* < 0.01).

**Table 2:** The correlations matrix of research variables

	1.	2.	3.	4.	5.	6.	7.	8.	9.	10.	11.	12.	13.	14.	15.
1.	1.000														
2.	−.010	1.000													
3.	.008	.093^b^	1.000												
4.	−.002	.116^b^	.254^b^	1.000											
5.	−.099^b^	.032^b^	.041^b^	.128^b^	1.000										
6.	.030^a^	.143^b^	.185^b^	.226^b^	−.132^b^	1.000									
7.	−.001	−.083^b^	−.116^b^	−.144^b^	−.144^b^	−.241^b^	1.000								
8.	<.001	−.073^b^	−.123^b^	−.138^b^	−.127^b^	−.221^b^	.967^b^	1.000							
9.	−.011	−.087^b^	−.111^b^	−.133^b^	−.133^b^	−.247^b^	.941^b^	.853^b^	1.000						
10.	.014	−.080^b^	−.068^b^	−.134^b^	−.166^b^	−.208^b^	.824^b^	.708^b^	.754^b^	1.000					
11.	.043^b^	−.029^a^	−.018	−.089^b^	−.139^b^	−.059^b^	.508^b^	.498^b^	.454^b^	.453^b^	1.000				
12.	.018	−.045^b^	−.072^b^	−.128^b^	−.153^b^	−.184^b^	.685^b^	.667^b^	.644^b^	.562^b^	.840^b^	1.000			
13.	.055^b^	.012	.069^b^	.014	−.042^b^	.147^b^	.009	.014	−.037^b^	.071^b^	.662^b^	.189^b^	1.000		
14.	.018	.109^b^	.094^b^	.183^b^	.160^b^	.441^b^	−.371^b^	−.318^b^	−.398^b^	−.357^b^	−.154^b^	−.319^b^	.159^b^	1.000	
15.	.017	.117^b^	.099^b^	.214^b^	.193^b^	.398^b^	−.533^b^	−.474^b^	−.552^b^	−.493^b^	−.255^b^	−.447^b^	.148^b^	.733^b^	1.000

1 = parents’ identity; 2 = relationship changes; 3 = physical exercise changes; 4 = dietary changes; 5 = Income changes; 6 = psychological stress due to COVID-19; 7 = resilience total; 8 = tenacity; 9 = strength; 10 = optimism; 11 = coping style total; 12 = positive coping; 13 = negative coping; 14 = anxiety; 15 = depression

^a^Correlation is significant at the 0.05 level

^b^0.01 level

### Regression analyses

Diagnostic tests indicated the absence of problematic multi-collinearity (tolerance values > 0.2 and variance inflation factor < 5) for all predictors. Detailed results of the four models are shown in Table 3. After adjusting the demographic variables, “parents who had a child with ASD” was associated with parental anxiety (*β* = 0.040, *P* < 0.001) and depression (*β* = 0.041, *P* < 0.001) scores in model 2. “Resilience” and “coping style” were significant predictors to anxiety (Δ*R*^*2*^ = 16.8%, *P* < 0.001) and depression (Δ*R*^*2*^ = 29.5%, *P* < 0.001). In model 3, “resilience” and “positive coping” were significantly negatively correlated with the scores of anxiety and depression (*β* = − 0.185, − 0.133 for anxiety; *β* = − 0.324, − 0.188 for depression), whereas “negative coping” showed a significant positive correlation (*β* = 0.173, 0.188, respectively). The COVID-19 crisis significantly influenced parents’ anxiety (Δ*R*^*2*^ = 12.9%, *P* < 0.001) and depression (Δ*R*^*2*^ = 7.3%, *P* < 0.001) scores. “Relationship changes”, “dietary changes” and “psychological stress” were significant predictors of anxiety scores (*β* = 0.034, 0.0468, 0.353), while “relationship changes,” “dietary changes” “income changes” and “psychological stress” were significant predictors of depression scores (*β* = 0.041, 0.088, 0.034, 0.242).

**Table 3:** The hierarchical linear regression analysis of independent variables correlated to scores of anxiety and depression in total participants (*n =* 6726)

	Anxiety			Depression		
	B (95%CI)	*β*	*P*	B (95%CI)	*β*	*P*
Model 1						
Province	**.587 (.347 ~ .826)**	**.058**	**<.001**	.107 (−.208 ~ .423)	.007	.505
Parents’ gender	.161 (−.298 ~ .621)	.007	.491	**1.035 (.431 ~ 1.640)**	**.032**	**.001**
Parents’ age	−.449 (−.727 ~ −.171)	−.035	.002	**−.861 (−1.227 ~ −.495)**	**−.048**	**<.001**
Parents’ health status	**2.942 (2.030 ~ 3.854)**	**.064**	**< 0.001**	**2.540 (1.340 ~ 3.741)**	**.039**	**<.001**
Parents’ education	**−.436 (−.663 ~ −.208)**	**−.044**	**.001**	**−.581 (−.880 ~ −.282)**	**−.041**	**<.001**
Parents’ occupation	.040 (−.127 ~ .208)	−.005	.639	.197 (−.023 ~ .418)	.017	.080
Child’s gender	−.272 (−.599 ~ .055)	−.016	.103	−.422 (−.853 ~ .008)	−.018	.054
Child’s age	**.423 (.178 ~ .668)**	**.042**	**.001**	.325 (.002 ~ .647)	.023	.048
Only child in the family	.406 (.054 ~ .758)	.025	.024	.603 (.140 ~ 1.065)	.026	.011
Parents’ marital status	−.127 (−.864 ~ .610)	−.003	.735	.316 (−.653 ~ 1.286)	.006	.522
Family income	−.200 (−.336 ~ −.064)	−.033	.004	−.255 (−.434 ~ −.076)	−.030	.005
*R*^*2*^	**.058**			**.077**		
Δ*R*^*2*^	**.058**			**.077**		
Model 2						
Group	**.739 (.357 ~ 1.122)**	**.040**	**<.001**	**1.101 (.597 ~ 1.604)**	**.041**	**<.001**
*R*^*2*^	**.061**			**.082**		
Δ*R*^*2*^	**.003**			**.005**		
Model 3						
Resilience total	**−.082 (−.094 ~ −.070)**	**−.185**	**<.001**	**−.205 (−.221 ~ −.188)**	**−.324**	**<.001**
Positive coping	**−.163 (−.198 ~ −.128)**	**−.133**	**<.001**	**−.326 (−.372 ~ −.281)**	**−.188**	**<.001**
Negative coping	**.290 (.256 ~ .324**	**.173**	**<.001**	**.447 (.402 ~ .493)**	**.188**	**<.001**
*R*^*2*^	**.229**			**.377**		
Δ*R*^*2*^	**.168**			**.295**		
Model 4						
Relationship changes	**.557 (.238 ~ .875)**	**.034**	**.001**	**.943 (.524 ~ 1.362)**	**.041**	**<.001**
Physical Exercise changes	−.241 (−.475 ~ −.007)	−.021	.043	−.452 (−.761 ~ −.144)	−.027	.004
Dietary changes	**.920 (.641 ~ 1.199)**	**.068**	**<.001**	**1.702 (1.334 ~ 2.069)**	**.088**	**<.001**
Income changes	.203 (−.038 ~ .444)	.019	.099	**.523 (.206 ~ .840)**	**.034**	**.001**
Psychological stress	**2.540 (2.390 ~ 2.690)**	**.353**	**<.001**	**2.474 (2.227 ~ 2.671)**	**.242**	**<.001**
*F*	**186.998**		**<.001**	**274.085**		**<.001**
*R*^*2*^	**.358**			**.450**		
Δ*R*^*2*^	**.129**			**.073**		

**Bold** = *P* < 0.001, Group: a healthy child = 0, an ASD child =1, *CI* confidence interval

With regard to anxiety scores, “resilience” and “coping strategy” explained 22% of the variance in ASD families, and 15% of the variance in TD families, after adjusting demographic variables. ASD families showed a greater variance in anxiety scores in terms of the impact of the COVID-19 crisis than TD families (Δ*R*^*2*^ = 13.9% vs. 12.7%, Table 4). While having a child with ASD was not associated with paternal anxiety scores, it was associated with maternal anxiety scores after adjusting demographic variables. The COVID-19 crisis could account for the higher variance in anxiety among mothers than fathers (Δ*R*^*2*^ = 13.1% vs. 1.14%, Table 5).

**Table 4:** The hierarchical linear regression analysis of independent variables correlated to scores of anxiety and depression in ASD and TD family

	Anxiety ASD family (*n =* 1764)			Anxiety TD family (*n =* 4962)			Depression ASD family (*n =* 1764)			Depression TD family (*n =* 4962)		
	B (95%CI)	*β*	*P*	B (95%CI)	*β*	*P*	B (95%CI)	*β*	*P*	B (95%CI)	*β*	*P*
Model 1												
Province	.242(−.280 ~ .763)	.021	.364	**.667(.409 ~ .946)**	**.071**	**<.001**	−.504(−1.155 ~ .146)	−.032	.129	.264(−.097 ~ .626)	.019	.151
Parents’ gender	.660(−.356 ~ 1.675)	.025	.203	.067(−.443 ~ .576)	−.003	.798	.687(−.580 ~ 1.954)	.019	.288	1.179(.494 ~ 1.864)	.038	.001
Parents’ age	−.307(−.921 ~ .307)	−.022	.327	−.501(−.810 ~ −.192)	−.042	.001	−.380(−1.146 ~ .386)	−.020	.331	**−1.006(− 1.421 ~ −.590)**	**−.058**	**<.001**
Parents’ health status	**2.877 (1.532 ~ 4.221)**	**.079**	**<.001**	**2.449 (1.137 ~ 3.761)**	**.043**	**<.001**	2.302(.625 ~ 3.980)	.046	.007	2.218(.453 ~ 3.983)	.027	.014
Parents’ education	−.768(−1.253 ~ −.283)	−.065	.002	−.325(−.582 ~ −.068)	−.035	.013	−.705(− 1.310 ~ −.100)	−.043	.022	−.512(−.858 ~ −.166)	−.038	.004
Parents’ occupation	−.306(−.670 ~ .059)	−.031	.100	.105(−.082 ~ .292)	.013	.272	.100(−.355 ~ .555)	.008	.665	.193(−.059 ~ .445)	.017	.134
Child’s gender	−.689(−1.445 ~ .067)	−.033	.074	−.188(−.546 ~ .169)	−.012	.302	−1.030(− 1.973 ~ −.086)	−.036	.032	−.278(−.759 ~ .203)	−.012	.256
Child’s age	.324(−.162 ~ .810)	.031	.191	**.486(.200 ~ .772)**	**.049**	**.001**	−.162(−.768 ~ .445)	−.011	.601	.511(.126 ~ .896)	.036	.009
Only child in the family	.280(−.451 ~ 1.011)	.015	.452	.412(.014 ~ .810)	.027	.042	.172(−.740 ~ 1.084)	.007	.712	.711(.176 ~ 1.246)	.032	.009
Parents’ marital status	−.258(−1.681 ~ 1.103)	−.007	.710	−.203(− 1.083 ~ .678)	−.005	.651	.079(−1.168 ~ 1.776)	−.002	.927	.210(−.974 ~ 1.395)	−.004	.728
Family income	.011(−.307 ~ .286)	−.002	.944	**−.278(−.430 ~ −.126)**	**−.050**	**<.001**	.220(−.150 ~ .590)	.023	.244	**−.412(−.617 ~ −.208)**	**−.051**	**<.001**
*R*^*2*^	**.068**			**.048**			**.072**			**.071**		
Δ*R*^*2*^	**.068**			**.048**			**.072**			**.071**		
Model 2												
Resilience total	**−.106(−.131 ~ −.080)**	**−.211**	**<.001**	**−.072(−.086 ~ −.058)**	**−.166**	**<.001**	**−.225(−.256 ~ −.193)**	**−.343**	**<.001**	**−.194(−.213 ~ −.175)**	**−.313**	**<.001**
Positive coping	**−.200(−.272 ~ −.129)**	**−.151**	**<.001**	**−.149(−.189 ~ −.110)**	**−.127**	**<.001**	**−.387(−.476 ~ −.297)**	**−.213**	**<.001**	**−.306(−.358 ~ −.253)**	**−.180**	**<.001**
Negative coping	**.333(.260 ~ .406)**	**.175**	**<.001**	**.276(.237 ~ .314)**	**.174**	**<.001**	**.461(.370 ~ .552)**	**.178**	**<.001**	**.444(.392 ~ .496)**	**.194**	**<.001**
*R*^*2*^	**.288**			**.198**			**.426**			**.347**		
Δ*R*^*2*^	**.220**			**.150**			**.353**			**.276**		
Model 3												
Relationship changes	.343(−.321 ~ 1.007)	.019	.311	.635(.275 ~ .995)	.041	.001	1.139(.311 ~ 1.967)	−.046	.007	.854(.396 ~ 1.338)	.038	.001
Physical Exercise changes	−.638(− 1.159 ~ −.116)	−.046	.017	−.110(−.369 ~ .149)	−.010	.404	**−.689(− 1.340 ~ −.039)**	**.036**	**.038**	−.359(−.708 ~ −.011)	−.023	.043
Dietary changes	**1.706 (1.101 ~ 2.311)**	**.109**	**<.001**	.653(.342 ~ .965)	.051	<.001	2.843 (2.088 ~ 3.598)	.133	<.001	**1.295(.876 ~ 1.715)**	**.070**	**<.001**
Income changes	.415(−.092 ~ .922)	.034	.109	.159(−.113 ~ .431)	.016	.252	.875(.243 ~ 1.508)	.053	.007	.437(.072 ~ .803)	.030	.019
Psychological stress	**2.884 (2.568 ~ 3.200)**	**.359**	**<.001**	**2.395 (2.227 ~ 2.564)**	**.351**	**<.001**	**2.890 (2.469 ~ 3.285)**	**.263**	**<.001**	**2.308 (2.081 ~ 2.534)**	**.235**	**<.001**
*F*	**68.610**		**<.001**	**125.138**		**<.001**	**100.843**		**<.001**	**182.347**		**<.001**
*R*^*2*^	**.428**			**.325**			**.523**			**.412**		
Δ*R*^*2*^	**.139**			**.127**			**.098**			**.065**		

**Bold** = *P* < 0.001, *ASD* autism spectrum disorder, *TD* typically developing, *CI* confidence interval

**Table 5:** The hierarchical linear regression analysis of independent variables correlated to scores of anxiety and depression in male and female participants

	Anxiety Fathers (*n =* 1017)			Anxiety Mothers (*n* = 5709)			Depression Fathers (*n* = 1017)			Depression Mothers (*n* = 5709)		
	B (95%CI)	*β*	*P*	B (95%CI)	*β*	*P*	B (95%CI)	*β*	*P*	B (95%CI)	*β*	*P*
Model 1												
Province	.467(−.188 ~ 1.123)	.047	.162	**.600(.341 ~ .859)**	**.059**	**<.001**	.046(−.839 ~ .931)	−.003	.919	.130(−.208 ~ .469)	.009	.451
Parents’ age	.173(−.519 ~ .866)	.015	.623	**−.557(−.862 ~ −.253)**	**−.043**	**<.001**	−.004(−.939 ~ .930)	.000	.993	**−1.024(−1.422 ~ −.626)**	**−.056**	**<.001**
Parents’ health status	1.775(−.177 ~ 3.728)	.048	.075	**3.246 (2.215 ~ 4.277)**	**.067**	**<.001**	.962(−1.674 ~ 3.598)	.018	.474	**2.921 (1.572 ~ 4.271)**	**.043**	**<.001**
Parents’ education	−.476(−1.033 ~ .081)	−.052	.094	−.439(−.688 ~ −.189)	−.043	.001	−.063(−.814 ~ .689)	−.005	.870	**−.692(− 1.018 ~ −.365)**	**−.048**	**<.001**
Parents’ occupation	.537(.068 ~ 1.005)	.059	.025	−.036(−.216 ~ .145)	−.004	.696	**1.145(.512 ~ 1.778)**	**.088**	**<.001**	.058(−.178 ~ .294)	.005	.629
Child’s gender	−.858(−1.698 ~ −.019)	−.053	.045	−.192(−.548 ~ .164)	−.012	.290	−1.045(−2.178 ~ .088)	−.045	.071	−.342(−.808 ~ .124)	−.015	.150
Child’s age	.137(−.471 ~ .744)	.015	.658	**.474(.206 ~ .742)**	**.046**	**.001**	−.016(−.836 ~ .804)	−.001	.970	.382(.030 ~ .733)	.026	.033
Only child in the family	.390(−.489 ~ 1.269)	.025	.385	.410(.025 ~ .794)	.025	.037	1.273(.087 ~ 2.460)	.056	.036	.498(−.005 ~ 1.002)	.021	.052
Parents’ marital status	−.963(−2.503 ~ .577)	−.034	.220	.213(−.630 ~ 1.055)	.005	.621	−.340(−2.419 ~ 1.738)	−.008	.748	.658(−.444 ~ 1.760)	.012	.242
Family income	−.022(−.352 ~ .309)	−.004	.898	−.234(−.384 ~ −.084)	−.038	.002	−.363(−.809 ~ .082)	−.049	.110	−.228(−.425 ~ −.032)	−.026	.023
*R*^*2*^	**.043**			**.060**			**.057**			**.072**		
Δ*R*^*2*^	**.043**			**.060**			**.057**			**.072**		
Model 2												
Group	.249(−.730 ~ 1.228)	.014	.618	**.844(.427 ~ 1.260)**	**.045**	**<.001**	1.380(.059 ~ 2.702)	.053	.041	**1.071(.526 ~ 1.616)**	**.040**	**<.001**
*R*^*2*^	.044			**.063**			**.066**			**.077**		
Δ*R*^*2*^	.001			**.003**			**.009**			**.005**		
Model 3												
Resilience total	**−.080(−.111 ~ −.049)**	**−.180**	**<.001**	**−.082(−.096 ~ −.068)**	**−.184**	**<.001**	**−.208(−.250 ~ −.166)**	**−.329**	**<.001**	**−.203(−.221 ~ −.185)**	**−.321**	**<.001**
Positive coping	**−.177(−.260 ~ −.094)**	**−.150**	**<.001**	**−.161(−.199 ~ −.123)**	**−.131**	**<.001**	**−.269(−.381 ~ −.157)**	**−.160**	**<.001**	**−.338(−.388 ~ −.288)**	**−.194**	**<.001**
Negative coping	**.301(.225 ~ .378)**	**.211**	**<.001**	**.288(.250 ~ .327)**	**.167**	**<.001**	**.461(.358 ~ .565)**	**.228**	**<.001**	**.447(.397 ~ .498)**	**.183**	**<.001**
*R*^*2*^	**.238**			**.227**			**.361**			**.375**		
Δ*R*^*2*^	**.194**			**.164**			**.295**			**.298**		
Model 4												
Relationship changes	.380(−.475 ~ 1.234)	.023	.383	.596(.252 ~ .940)	.037	.001	.495(−.659 ~ 1.648)	.021	.400	**1.007(.557 ~ 1.457)**	**.044**	**<.001**
Physical Exercise changes	−.094(−.658 ~ .470)	−.009	.744	−.270(−.527 ~ −.012)	−.023	.040	−.388(−1.149 ~ .373)	−.026	.318	−.466(−.803 ~ −.129)	−.028	.007
Dietary changes	.732(.041 ~ 1.424)	.058	.038	**.960(.655 ~ 1.266)**	**.070**	**<.001**	1.174(.241 ~ 2.108)	.065	.014	**1.802 (1.402 ~ 2202)**	**.092**	**<.001**
Income changes	.331(−.264 ~ .926)	.033	.275	.185(−.079 ~ .449)	.017	.170	.825(.022 ~ 1.629)	.058	.044	.508(.162 ~ .854)	.033	.004
Psychological stress	**2.284 (1.901 ~ 2.667)**	**.330**	**<.001**	**2.574 (2.411 ~ 2.737)**	**.355**	**<.001**	**1.997 (1.480 ~ 2.513)**	**.204**	**<.001**	**2.540 (2.326 ~ 2.753)**	**.248**	**<.001**
*F*	**28.532**		**<.001**	**167.260**		**<.001**	**36.951**		**<.001**	**246.761**		**<.001**
*R*^*2*^	**.352**			**.358**			**.413**			**.452**		
Δ*R*^*2*^	**.114**			**.131**			**.052**			**.077**		

**Bold** = *P* < 0.001; Group: a healthy child = 0, an ASD child =1, *CI* confidence interval

With regard to depression scores, “resilience” and “coping strategy” explained 35.3% of the variance in ASD families, and 27.6% of the variance in TD families, after adjusting demographic variables. ASD families showed a higher variance in depression scores in terms of the impact of the COVID-19 crisis than TD families (Δ*R*^*2*^ = 9.8% vs. 6.5%, Table 4). While having a child with ASD was not associated with paternal depression scores, it was associated with maternal depression scores after adjusting demographic variables. The COVID-19 crisis could account for the higher variance in depression among mothers than fathers (Δ*R*^*2*^ = 7.7% vs. 5.2%, Table 5).

## Discussion

With the implementation of stringent restrictions in some countries, the COVID-19 pandemic has presented unprecedented challenges for parents in terms of home quarantine, remote working and continuous parenting for long periods of time. The gradual devotion of so much time to parenting and caregiving has brought parents to the brink of collapse, and families who have children with ASD may face even greater challenges.

### Effect of having a child with ASD on parental anxiety and depression

As we all know, having a child with ASD is a significant life event that may provoke existential questions and dilemmas for parents. These parents experienced a complex array of stressors over time, which included: (a) the severity of the child’s ASD-related symptoms; (b) sustained time pressures; (c) changing life plans; (d) persistent vigilant parenting; (e) cost of care; (f) discrimination; and (g) inadequate social support. These stressors caused the parents of children with ASD to feel less confident and optimistic in their daily lives, and they reported that they experienced greater anxiety and depression symptoms [12]. In fact, 80% of parents of children with ASD claimed that they sometimes felt “stretched beyond their limits” [21], and 12% of parents of children with ASD were in the clinical range for anxiety symptoms, while 25% reported clinically-significant depression symptoms [22]. Under normal circumstances, researchers identified elevated levels of parental anxiety and depression among parents of children with ASD, in comparison with both parents of TD children and parents of children with other developmental disabilities [12, 23, 24]. Our results revealed that raising a child with ASD was a significant independent factor that contributed to parental anxiety and depression symptoms. In accordance with our finding, Sharpley et al. indicated that coping with a child’s behavioral problems was the biggest contributor to anxiety and depression among parents of children with ASD [21].

### Effect of resilience and coping strategies on parental anxiety and depression

Once faced with an unexpected event, parents of children with ASD are more likely to be influenced by external factors and unable to respond with positive resilience and coping strategies, which results in poor psychological outcomes. Thus, this perhaps leads to a self-perpetuating cycle of parental anxiety and depression. The present study found that parents of children with ASD had lower levels of resilience and were more likely to adopt negative rather than positive coping strategies compared with parents of TD children during the COVID-19 pandemic. This result was in accordance with the findings of Lai et al. [25], which demonstrated that parents of children with ASD used in more maladaptive coping strategies than parents of TD children in the ordinary period. Our results also confirmed that resilience and coping strategies were significant independent predictors of anxiety and depression symptoms. Lower resilience, less positive coping strategies, and more negative coping strategies were linked to anxiety and depression symptoms. These findings are consistent with previous research [25–27]. Although resilience and coping strategies were not completely identical to parenting stress, they provided a holistic understanding of the stress experienced by parents of children with ASD.

### Effect of COVID-19 pandemic on parental anxiety and depression

During the COVID-19 pandemic, parents emerged with various types of emotional problems, to the extent that some parents experienced symptoms of anxiety and depression which included washing their hands frequently and finding themselves preoccupied with physical discomfort. The present study showed that 6.6 and 21.7% of parents of TD children suffered from anxiety and depression symptoms, respectively, which was similar to the results observed in the case of Chinese workers (the age range of Chinese workers was similar to the age range of parents in the current study) [28]. Zhang et al. [28] indicated that 3.4 and 22.8% of Chinese workers exhibited anxiety and depression symptoms. Furthermore, as expected, more parents of children with ASD experienced anxiety and depression symptoms during the COVID-19 pandemic (i.e., 12.2 and 31%, respectively). In respect to anxiety symptoms, no obvious differences were observed in parents of TD children in this study, as well as among the general population before the COVID-19 outbreak in China [29]. In addition, we found a relatively high rate of depression symptoms in parents of children with ASD, compared to others surveys that used different populations during the corresponding period [28, 30, 31]. According to the Global Burden of Disease in 2017, The rates of depression symptoms in parents of TD children and in parents of children with ASD in the present study were obviously higher than those observed among the general population (Heilongjiang: 4.25%, Henan:3.89%, Fujian:3.83%) [32]. The results of the present study revealed that anxiety and depression symptoms were attributable to the COVID-19 pandemic itself, the rates of which amounted to 12.9 and 7.3%, respectively, among the total participants. The psychological stress brought about by the COVID-19 pandemic played the most important role in parental anxiety (*β* = 0.353) and depression (*β* = 0.242) scores. A range of psychiatric morbidities were reported during the early phase of the SARS outbreak, including persistent depression, anxiety, panic attacks, psychomotor excitement, psychotic symptoms, delirium, and even suicidality [33]. The physical harm caused by such public health emergencies may be recoverable in the short term, but the psychological harm is far-reaching. Considering the overwhelming worldwide transmission that has occurred and the rapidly-evolving situation of COVID-19, which is a serious global health catastrophe, its impact is certainly more severe than that of the SARS outbreak in 2003. Unsurprisingly, the present study further demonstrated the relationship between the COVID-19 pandemic and the increase in symptoms of anxiety and depression which reached 13.9 and 9.8%, respectively, among parents of children with ASD. Obviously, parents of children with ASD were more vulnerable to the COVID-19 crisis than parents of TD children. The immense impact of having a child with autism is evident in terms of both the severity and the breadth of parental and family domains that appear to be affected, especially in the course of COVID-19 pandemic.

### Gender differences in parental anxiety and depression

Notably, in light of the different gender roles in the family, gender differences emerged as a factor that influenced anxiety and depression symptoms. Generally speaking, mothers, who are usually the primary caregivers of their children, experienced higher levels of parental stress and mental problems. Among the entire sample in the present study, mothers showed higher values of PSCQ compared with fathers (10.3 ± 7.7 vs. 9.2 ± 7.2, *P* = 0.004, data not shown), and similar trends were observed in the case of the symptoms of anxiety and depression. In the case of ASD families, this study found similar results, indicating that more mothers than fathers of children with ASD suffered from anxiety (12.6% vs. 10.2%) and depression (31.7% vs 26.4%) problems. Western and eastern research, along with the current study, offer support for the notion that mothers of children with ASD experience greater stress, anxiety and depression compared with fathers [34–36]. Moreover, the current study found that raising a child with ASD was a significant factor with respect to anxiety and depression symptoms among mothers, but this was not the case for fathers, and paternal anxiety symptoms were not associated with the child’s characteristics. Therefore, although mothers and fathers of children with ASD bear similar burdens together, mothers are faced with particularly heavy responsibilities in terms of the level of care that is required when raising a child with ASD. The relationship between parents’ mental health (anxiety and depression) and raising a child with autism is a complex, transactional process. Parents’ adverse emotional experiences, in turn, undermined the closeness of the parent-child relationship, diminished the efficiency of parenting and well-being, and further exacerbated ASD-related behaviors and symptoms [37]. Studies have also shown that higher levels of anxiety and depression in parents are associated with lower expectations regarding treatment, which means that greater obstacles are encountered during treatment, and earlier cessation of treatment can occur [12].

### Precautionary measures to address psychological distress during COVID-19 pandemic

Family training seems to be an impressive remedy to alleviate the emotional burdens and practical demands of the entire ASD family system during the COVID-19 pandemic. On this occasion, above all, the Chinese parents of children with ASD should move away from the traditional notion which involves excessively parental reliance on educational institutions, to the extent that the role of the family in rehabilitation is underestimated. In the long run, it would be beneficial to develop a parental training program to improve parents’ mental health and parent-child interaction [13, 38]. Moreover, measures such as financial assistance, the establishment of a joint community-hospital-family rehabilitation system, and a vocational protection policy can also be undertaken by government agencies and ASD rehabilitation institutions to improve the mental health status of parents.

### Limitations

The data in this study were collected from Heilongjiang, Henan, and Fujian provinces, which are located in northern, central and southern of China, and these regions are associated with different cultural and economic situations which enhanced the generalizability of this research. Nonetheless, there were several potential limitations in the present study. First, this study was a cross-sectional survey and it could not capture the changes in psychological distress over the course of the COVID-19 pandemic. Moreover, it is very hard to meaningfully disentangle the effects of ASD itself from the effect of COVID-19. Second, since the survey method was based on network and convenience sampling, the severity of a child’s ASD-related symptoms and the psychiatry or psychological conditions of TD children in the regular schools could not be controlled to minimize the influence of demographic variables. Third, non-parent participants (grandparents) were ruled out due to the adequate sample size. Future studies could employ a longitudinal design and carry out in-depth interviews.

## Conclusion

The COVID-19 pandemic caused parents to suffer from varying levels of psychological distress. The parents of children with ASD exhibited higher levels of psychological distress, and anxiety and depression problems were more prominent. The accumulation of multiple undesirable factors has prevented parents of children with ASD from responding positively to the COVID-19 crisis. During this unprecedented public health crisis, attention should not only be paid to the development of children with ASD, but to the mental health of their parents. The development and implementation of mental health assessments, supports, treatments and services for parents of children with ASD are also crucial responses to the COVID-19 crisis.

## Supplementary Information

**Additional file 1: Appendix A**. The psychological stress from COVID-19 questionnaire. **Table S1**. The variables’ values assigned in the regression analyses.

## Data Availability

The dataset used during the current study is available from the corresponding author on reasonable request.
