# Supplementary material for: The relationship between 2019-nCoV and psychological distress among parents of children with autism spectrum disorder
Source: Global Health. 2021 Feb 25;17:23. doi: 10.1186/s12992-021-00674-8 (PMC7905970; doi:10.1186/s12992-021-00674-8)
Supplement: Supplementary file 1 — Additional file 1: Appendix A. The psychological stress from COVID-19 questionnaire. Table S1. The variables’ values assigned in the regression analyses. [file 12992_2021_674_MOESM1_ESM.docx]

Appendix 1. The Psychological Stress from COVID-19 Questionnaire.

| Main question | Psychological Stress from COVID-19 Questionnaire | Options |
| --- | --- | --- |
| Depending on your behaviors and perceptions during the COVID-19 pandemic, choose the option that best suits you. | 1. You worried about being infected with yourself and your family. | 0 = Not at all  1 = Infrequently  2 = Moderately frequently  3 = Very frequently |
|  | 2. Your words were less than usual. |  |
|  | 3. You washed your hands and scrubbed things over and over again, but always felt not clean enough. |  |
|  | 4. You had too much worry about all kinds of things. |  |
|  | 5. You doubted yourself to have been infected, filled your head with nonsense and could not control. |  |
|  | 6. You felt no spirit, trouble in concentrating on and remembering things, and your mind going blank. |  |
|  | 7. Even with the help from your lovers or friends, you could not get rid of your depression. |  |
|  | 8. You had no interest in anything, and no energy in doing things. |  |
|  | 9. Referring to things related to the COVID-19 pandemic, you felt scared and the heart beat faster. |  |
|  | 10. You felt uneasy or tense in places where the crowds were gathered, especially near the hospital. |  |
|  | 11. You always had an ominous hunch. |  |
|  | 12. You felt yourself useless or worthless. |  |
|  | 13. You cared excessively about any physical discomfort. |  |
|  | 14. You did not go to the hospital or the crowd gathered place, always wore a mask when in contact with people. |  |
|  | 15. When you thought of something related to the COVID-19 pandemic, you did not have the mind to do anything else. |  |

Table S1 The variables’ values assigned in the regression analyses.

| Variables | Code |
| --- | --- |
| Province |  |
| Heilongjiang | 1 |
| Henan | 2 |
| Fujian | 3 |
| Parents’ gender |  |
| Male | 1 |
| Female | 2 |
| Parents’ age (years) |  |
| 20~30 | 1 |
| 31~40 | 2 |
| 41~50 | 3 |
| 51~60 | 4 |
| Parents’ health status |  |
| Well | 1 |
| Diseased | 2 |
| Parents’ education |  |
| Secondary school or below | 1 |
| High school or same level | 2 |
| College or same level | 3 |
| Postgraduate | 4 |
| Parents’ occupation |  |
| Manual workers | 1 |
| Mental workers | 2 |
| Unemployed | 3 |
| Others | 4 |
| Child’s gender |  |
| Male | 1 |
| Female | 2 |
| Child’s age (years) |  |
| Up to 3 | 1 |
| 3~ 6 | 2 |
| 6~12 | 3 |
| 12~18 | 4 |
| Only child in the family |  |
| Yes | 1 |
| No | 2 |
| Parents’ marital status |  |
| Married | 1 |
| Divorced or widowed | 2 |
| Family income per month |  |
| <3000 | 1 |
| 3000~6000 | 2 |
| 6001~9000 | 3 |
| 9001~12000 | 4 |
| 12001~15000 | 5 |
| >15000 | 6 |
| group |  |
| Typically developing children | 0 |
| Autism spectrum disorder | 1 |
| Parents’ identity |  |
| General public | 1 |
| Quarantine | 2 |
| Front-line staff | 3 |
| Relationship changes |  |
| Increased | 1 |
| No change | 2 |
| Decreased | 3 |
| Physical Exercise changes |  |
| Better | 1 |
| No change | 2 |
| Worse | 3 |
| Dietary changes |  |
| Better | 1 |
| No change | 2 |
| Worse | 3 |
| Income changes |  |
| Normal income | 1 |
| Partial income | 2 |
| No income | 3 |
| Psychological stress |  |
| Low | 1 |
| Relatively low | 2 |
| Relatively high | 3 |
| high | 4 |
